# Ecological Momentary Assessment of Masseter Muscle Activity in Patients with Bruxism

**DOI:** 10.3390/ijerph20010581

**Published:** 2022-12-29

**Authors:** Diana Elena Vlăduțu, Mihaela Ionescu, Răzvan Mercuț, Lorenzo Noveri, Grigore Lăzărescu, Sanda Mihaela Popescu, Monica Scrieciu, Horia Octavian Manolea, Monica Mihaela Iacov Crăițoiu, Alin Gabriel Ionescu, Veronica Mercuț

**Affiliations:** 1 Department of Prosthetic Dentistry, University of Medicine and Pharmacy of Craiova, 200349 Craiova, Romania; 2 Department of Medical Informatics and Biostatistics, University of Medicine and Pharmacy of Craiova, 200349 Craiova, Romania; 3 Department of Plastic Surgery, University of Medicine and Pharmacy of Craiova, 200349 Craiova, Romania; 4 Studio Dentistico Noveri-Minelli, 18038 Sanremo, Italy; 5 Department of Oral Rehabilitation, University of Medicine and Pharmacy of Craiova, 200349 Craiova, Romania; 6 Department of Dental Materials, University of Medicine and Pharmacy of Craiova, 200349 Craiova, Romania; 7 Department of Medical History, University of Medicine and Pharmacy of Craiova, 200349 Craiova, Romania

**Keywords:** bruxism, muscular contractions, masseter muscle, clenching, grinding, EMG

## Abstract

According to the International Bruxism Consensus, bruxism refers to the activity of the masticatory muscles reflecting contraction disorders, regardless of whether it is during sleep (SB) or an awake (AB) state. The objective of the present study was to evaluate the activity of the masseter muscle by surface electromyographic (sEMG) recordings. This study was performed on 20 participants with self-reported “possible bruxism” (study group) and 20 participants with no self-reported bruxism (control group); all participants underwent an evaluation of the masseter muscle activity using the dia-BRUXO device, which provides numerical parameters regarding sEMG (the total duration and the type of bruxism specific events, the effort made by the masticatory muscles during the recording period, and the personal bruxism index of each participant). Participants from the study group presented more clenching events during AB, three times more frequent than the control group (*p* = 0.002, Mann–Whitney U test); for SB, the frequency of clenching and grinding events was comparable within the study group, being more frequent than for the control group; the mean value of the effort index was higher for AB (1.177%) than SB (0.470%) and the same for the duration index, with a mean value of 2.788% for AB and 1.054% for SB. All participants from the control group presented reduced values for all acquired parameters. Overall, the personal bruxism index in AB was approximately four times higher for the study group (2.251%) compared to the control group (0.585%) (*p* < 0.005, Mann–Whitney U test). Similar values were obtained for SB. All participants with “possible bruxism” from the study group presented a higher activity of the masseter muscle, which is specific for bruxism, thus being defined as “definite bruxism”.

## 1. Introduction

The term “bruxism” was used for the first time in literature in 1931 by Frohman, to denote “parafunctional clenching or grinding of the teeth” [1,2], referring specifically to the contacts between the teeth of the two arches. Over time, several other definitions have been proposed to better reflect the elements involved in the generation of bruxism and its manifestations.

In 2012, following a consensus reunion of experts in bruxism [3], a new definition was elaborated referring to the masseter muscles’ activity, which generates clenching, grinding, rigidity, and pushing of the mandible, both in sleep bruxism (during nighttime—SB) as well as in awake bruxism (during daytime). This definition was based on the previous definitions defined in Orofacial Pain Guidelines (OFPG-4), fourth edition [4], in the International Classification of Sleep Disorders (ICSD-2), second edition [5], as well as in the Glossary of Prosthodontic Terms (GPT-8) [6].

Together with this definition, a new diagnostic system was also proposed. Considering that the gold standard for sleep bruxism is represented by polysomnography and the results of current instrumental examinations are interpretable, a gradual diagnostic system for SB and AB was proposed: possible, probable, and definite bruxism [3]. The experts who proposed this gradual system considered that possible SB or AB should be based on self-report, on questionnaires, and/or anamnesis. Probable SB or AB bruxism should be diagnosed through self-report and clinical examination. The diagnosis of definite bruxism should be based on self-report, with or without clinical examination, and polysomnographic recordings, preferably associated with supplementary audio-video recordings.

In 2017, a new consensus meeting reunited experts in bruxism, prior to the 95th General Session & Exhibition of the International Association for Dental Research (IADR) [7], when separate definitions were defined for SB and AB. Both types of bruxism represent masticatory muscle activities; however, during sleep, the activity is either non-rhythmic (tonic) or rhythmic (phasic) and it is not a sleep disorder, while during wakefulness, the activity is defined by sustained or by repetitive tooth contact, associated or not with bracing or thrusting of the mandible. In both cases, the activity is not a movement disorder but the activity of the masticatory muscles, emphasizing the fact that the contraction disorders of the masticatory muscles are at the origin of bruxism, regardless of whether it is during a sleep or awake state. Furthermore, both definitions end with the fact that the individuals are healthy, which indicates that, for most persons, bruxism is not a disorder but a sign of an affection or a risk factor. During this second meeting, the diagnosis system proposed in 2012 was re-evaluated, to define the reliability, sensibility, and specificity of every source of information.

Due to the limits related to polysomnography, in 2019, experts who developed the International Consensus regarding bruxism [8] drew attention to the limits of instrumental evaluations by polysomnography [8,9]. Therefore, a directive-document was published in 2020 regarding the creation of a Standardized Tool for the Assessment of Bruxism (STAB) [8,9,10,11,12,13].

According to this document, future research will be redirected on two different axes: axis A will include studies that evaluate bruxism, self-reporting, clinical evaluation, and instrumental evaluation, while axis B concerns studies regarding bruxism etiology, risk factors, and concomitant affections. As part of future research, specific instruments for bruxism evaluation will be selected and clinically tested in order to include them in the extended version of the general bruxism evaluation system. Its purpose is to differentiate bruxism episodes by other motor activities of the masticatory muscles, especially for EMG recordings performed in the patient’s natural environment [11,14]. In addition, according to this document, the gold standard should be based on the evaluation of masticatory muscle activity by polysomnography or electromyography, but ecological momentary evaluation (EMA) can be a useful instrumental evaluation method, with portable electromyographs, even with a single recording channel [11,12].

All studies based on instrumental evaluations should use validated devices when they are available. If not, specific equipment will be developed for this purpose [11]. The dia-BRUXO device is dedicated to this purpose, and its usage is encouraged in bruxism studies. Bracci et al. [12] presents the advantages of this device and emphasizes them using EMG recordings.

The objective of the present study is to highlight, by surface electromyographic (sEMG) recording of the masseter muscle, a pattern of muscle activity in students with bruxism, to assess the activity that characterizes the masticatory muscles and to establish the diagnosis of “definite bruxism” based on these recordings.

## 2. Materials and Methods

### 2.1. Participant Selection Process

The present research was carried out during 2022 and is the continuation of the study carried out in 2021 on 328 students with ages within the interval of 21–41 years old, of both sexes, from the Faculty of Dental Medicine, University of Medicine and Pharmacy of Craiova, Romania [10]. In this initial study based on a questionnaire, the diagnosis of “possible bruxism” was established for 129 students, while the remaining 199 students reported no signs of bruxism. The questionnaire included questions regarding epidemiological data but also the presence of bruxism episodes, while aiming to determine an association with stress, sleep disorders (insomnia), anxiety, and manifestations of temporomandibular disorder.

From the previous study, 20 participants who were classified as presenting “possible bruxism” associated with stress and bruxism manifestations formed the study group, and 20 participants who did not present signs of bruxism formed the control group. The inclusion criteria were the existence of informed consent for data recording and processing and residence in Craiova during the recording period.

The exclusion criteria specific to this study were the following: the existence of edentulous spaces or prosthetic restorations, periodontal diseases, active pathological processes in the masticatory system, the presence of orthodontic appliances, and the presence of general conditions that require the administration of anti-inflammatory, sedative, or relaxing muscular drugs. In addition, during the 24-h recording interval, the evaluated participants did not consume alcohol, tobacco, or coffee.

The 40 participants underwent sEMG recordings of the masseter muscle using the dia-BRUXO electromyograph for 24 h in their natural environment.

Before the actual recording for each participant, an electronic document was filled in with personal data (name, surname, age, gender, domicile) and several specific manifestations of bruxism (tooth wear, abfraction lesions, masticatory muscle pain, temporomandibular joint (TMJ) pain, headache and neck muscle pain, snoring, and whether the patient wears a mouth guard or not).

### 2.2. sEMG Recordings

The sEMG recordings of the masseter muscle were made using the dia-BRUXO device.

The dia-BRUXO electromyograph is manufactured by Biotechnovations S.R.L., based in Via G. Matteotti, 189-18038 Sanremo IM, Italy. Its dimensions are 43 × 50 × 10 mm, and it weighs 16 g. The device is equipped with two metal inserts inside, corresponding to the disposable electrodes provided, a lithium battery, and a single recording channel.

The dia-BRUXO device is applied under the left earlobe, with the electrodes positioned anterior to the ear on the cutaneous projection of the masseter muscle.

The device was calibrated at the beginning of the recordings for each participant. Thus, after applying the device, the participant of the study positioned the mandible for one minute in the mandibular resting position (posture position), in the centric occlusion position (performed a few swallowing movements), and performed maximum contractions for one minute.

The recordings were made starting in the morning until the same time the following day. Thus, approximately 24 h of recording data were obtained for each participant. At the end of this period, the device provided a report regarding the sleep period and the awake period. Additionally, the device reported the detachment period, which represents that the ensemble of time intervals when the electrodes were no longer properly attached to the participant’s skin.

The dia-BRUXO device provided the following variables:

1. *T*: total duration of the period in which data were acquired;
2. *t*: the time interval between the reception of two events, in this case, 100 mS;
3. *n*: total number of recorded data points;
4. *i*: reference number between 1 and n (inclusive);
5. *x_i_*: value of data recorded by sEMG (in μV);
6. *x_max_*: maximum value recorded by sEMG (in μV);
7. *k_i_*: binary value related to recorded data.

For ease of calculations, if “*i*” corresponds to a parafunctional activity, set *k_i_* = 1, otherwise = 0.

With the help of these variables, the device exported the following computed parameters: BTI—Bruxism Time Index, BWI—Bruxism Effort Index, and BPI—Personal Bruxism Index.

### 2.3. Bruxism Time Index

BTI represents the percentage of time in which a person is experiencing bruxism episodes from the total recording period. This parameter is used to compare bruxism activity for different persons.

(1) $$\mathrm{BTI}=\frac{100\times t\sum_{i=1}^{n}k_{i}}{1000\times T}$$

(2) $$\mathrm{BTI}=\frac{t\sum_{i=1}^{n}k_{i}}{10\times T}$$

The sum from the numerator in the first formula is used to compute the recording data regarding bruxism episodes during the entire evaluation period. By multiplying this number with *t*—in the above example, it is 100 mS—we obtain the total time of bruxism episodes, expressed in mS.

To convert the effective recording duration, the denominator is multiplied by 1000, and to convert the entire fraction to percentages, the numerator is multiplied by 100, obtaining Equation (2).

BTI values close to 0% indicate that the person has rare bruxism episodes, while high values indicate that the person presents frequent bruxism episodes.

MTI represents a similar index but computed for the masseter muscle.

### 2.4. Bruxism Effort Index

BWI represents a percentage from the entire effort corresponding to the activity of the masseter muscle during bruxism episodes, compared to the maximum effort that the muscle could experience in the same time interval. The computation of this percentage allows maintenance of flexibility, variability, and the possibility to compare various cases, without compromising the data validity.

The formula to compute the bruxism effort index is:

(3) $$\mathrm{BWI}=\frac{100\times t\sum_{i=1}^{n}x_{i}k_{i}}{\sum_{i=1}^{n}x_{max}k_{i}}$$

Due to the operator *k_i_* from this formula, all recorded data, except the bruxism episodes, are ignored, as their value is multiplied by zero. In this way, the numerator from Equation (3) computes the sum of all EMG values reordered during bruxism episodes, while the denominator computes a similar sum, only that the values of *x_i_* are replaced by the values of *x_max_*. The result is then multiplied by 100 to obtain percentage values.

BWI values close to 0% indicate a person with bruxism episodes by a low amplitude contraction of the masseter muscles, while increased values indicate a person with bruxism episodes characterized by contractions of the masseter muscles close to the maximum effort capacity.

MWI represents a similar index but computed for the masseter muscle.

### 2.5. Personal Bruxism Index

BPI represents a parameter that describes the degree of bruxism specific to a person, such as various cases which may be ordered according to their severity. This index depends on bruxism’s duration and intensity and, eventually, on other factors such as a person’s symptoms.

BPI’s formula takes into account the following parameters:

(4) BPI=αBTI+βBWI

where *α* and *β* belong to R, such as:

(5) α+β=1

This formula represents the weighted average of factors describing the severity of bruxism.

The proposed formula for BPI is:

(6) $$\mathrm{BPI}=\frac{2}{3}\mathrm{BTI}+\frac{1}{3}\mathrm{BWI}$$

The fact that BTI has a double weight compared to BWI is based on clinical observed values.

MPI represents a similar index but computed for the masseter muscle.

### 2.6. Statistical Analysis

Statistical analysis was completed using the SPSS (Statistical Package for Social Sciences) software, version 20 (SPSS Inc., Chicago, IL, USA). Continuous variables were given as the pair “mean ± standard deviation” (SD), while nominal and ordinal variables were described as frequency distributions and percentages. The Kolmogorov–Smirnov/Shapiro–Wilk test was used to evaluate the normal distribution of the variables acquired in this study. According to the results obtained, comparisons between groups with and without “possible bruxism” were performed with the t-Student test for normally distributed data as well as with the Mann–Whitney U test for the other variables. The chi-square test was applied to categorical data. All *p* values less than 0.05 reflected statistically significant results.

To carry out this study, informed consent was obtained from all study participants regarding the recording, processing, and publication of data, in accordance with the Declaration of Helsinki. Before the start of the study, the approval of the Ethics Commission of the University of Medicine and Pharmacy of Craiova was obtained (no. 84/03.06.2021).

## 3. Results

### 3.1. Characteristics of the Study Groups

After the inclusion and exclusion criteria were applied, the study group of 20 participants with “possible bruxism” and the control group of 20 participants without the self-reported bruxism were formed. From the 40 participants that were included in the study, 30 were females (75%) and 10 were males (25%), with the age between 20 and 53; however, most of the participants were in their 20s, such that the overall mean age was 27 ± 6.85 years. The gender distribution between the two groups was identical. Moreover, there was no statistical difference between the mean ages of the participants, *p* > 0.05. The characteristics of the participants are presented in Table 1.

### 3.2. sEMG Evaluation Results

The data recorded by the dia-BRUXO device for 24 h were transferred to a personal computer, and the recordings were displayed in PDF format. The sEMG events recorded in this research were classified into several types of activities specific to bruxism, and their intensity was measured in μVrms.

**Figure 1:**
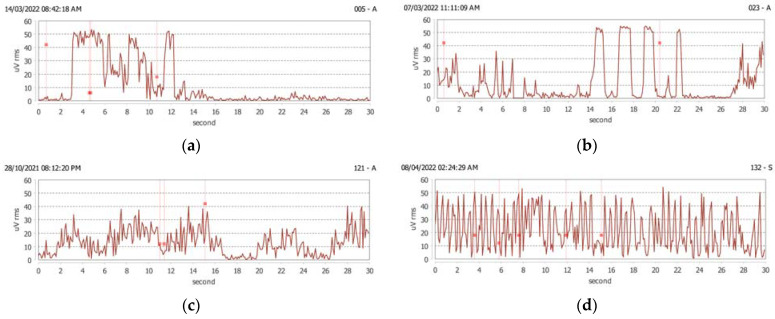

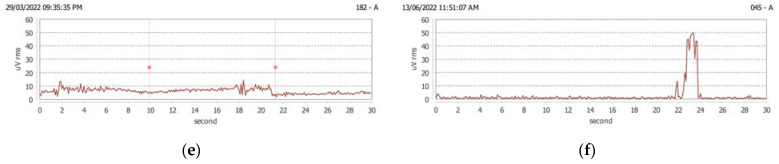
sEMG events (expressed by root–mean–square voltage at a specific moment of time): (**a**) tonic contraction, (**b**) rhythmic or phasic contraction, (**c**) light grinding or rubbing of the teeth, (**d**) severe grinding, (**e**) mandibular clenching, and (**f**) pushing the mandible.

*Tonic contraction*—EMG of the masseter muscle showed an activity ≥ 36 μVrms, prolonged for ≥ 2 s (Figure 1a).

*Rhythmic or phasic contraction*—EMG of the masseter muscle showed rhythmic and repetitive activity ≥ 36 μVrms (from 2 to 4 episodes every 6 s) in which each episode had a duration of ≥ 0.5 s (Figure 1b).

*Light grinding* or rubbing of the teeth—EMG of the masseter muscle showed an activity between 10 and 30 μVrms and continued for at least 4 s (Figure 1c). It represents light contact between the teeth with protrusive–retrusive friction of the teeth and/or sides or combined movements.

*Severe grinding*—EMG of the masseter muscle showed significant activity between 20 and 40 μVrms and continued for at least 4 s (Figure 1d).

*Bracing of the mandible*—EMG of the masseter muscle showed isotonic and isometric activity between 4 and 12 μVrms (such as that occurring in speech) (Figure 1e).

*Thrusting of the mandible*—EMG of the masseter muscle shows little or no activity, because the masseter muscle is very little involved (Figure 1f).

**Figure 2:**
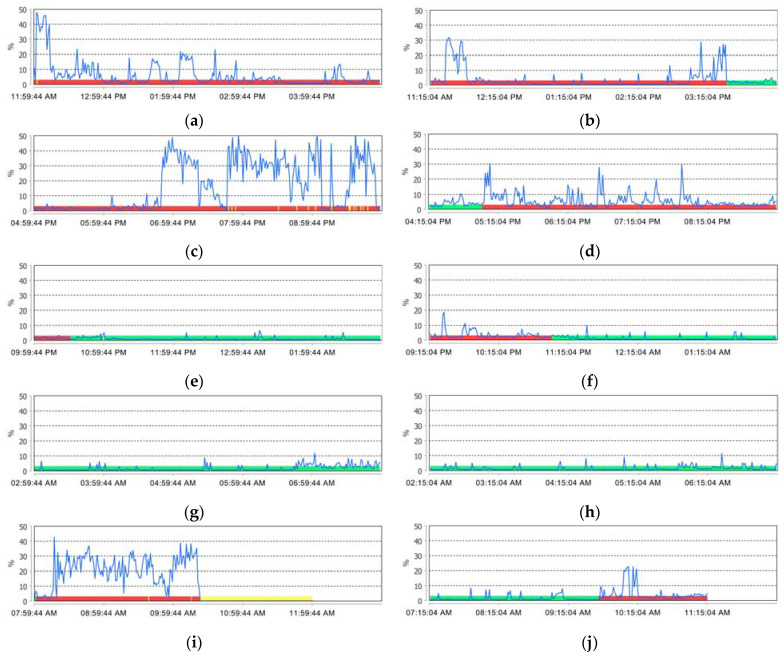
Comparative analysis of the summary of relevant activities recorded at the masseter level (sleep time interval—green color, awake time interval—red color, detachment time interval—yellow color) for: (**a**,**c**,**e**,**g**,**i**) one participant from the study group and (**b**,**d**,**f**,**h**,**j**) one participant from the control group.

Based on all the recordings, the device made a summary of the relevant activities recorded at the masseter level, which were reported as a percentage of the maximum contraction (Figure 2a–j).

Analyzing the data exported by the device, the following were found: the duration of the recordings varied from 15 h to 24 h, and most participants (87.5%) had recordings lasting 24 h. The duration of the sleep period was between 19.20% and 50.70% of the recorded period for all participants, with a mean of 32.64%, without statistically significant differences between the two groups of participants (Table 2). The duration of the awake period was between 49.30% and 80.80% of the recorded period for all participants, with a mean of 67.07%, and no statistically significant differences were determined between the two groups of participants (Table 2).

The duration of electrode detachment varied between 0.00% (for 62.50% of all 40 participants) and 17.40% of the recorded period, with a mean of 0.98%.

### 3.3. Evaluation of Recorded Activities in Bruxism

Regarding the type of events recorded, the dia-BRUXO device provided data on the number of clenching events, the number of grinding events, and the number of other events recorded.

The control group presented a lower number of events for all three categories, the differences between the groups being statistically significant (Table 3).

**Figure 3:**
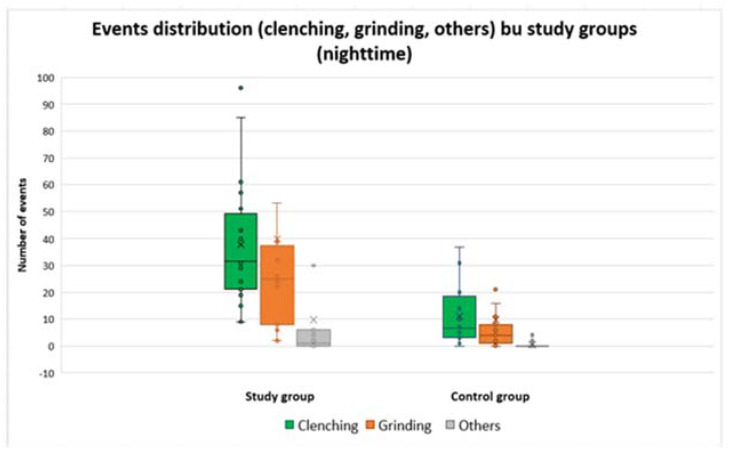
Distribution of clenching, grinding, and other types of events, for the two groups, during nighttime.

**Figure 4:**
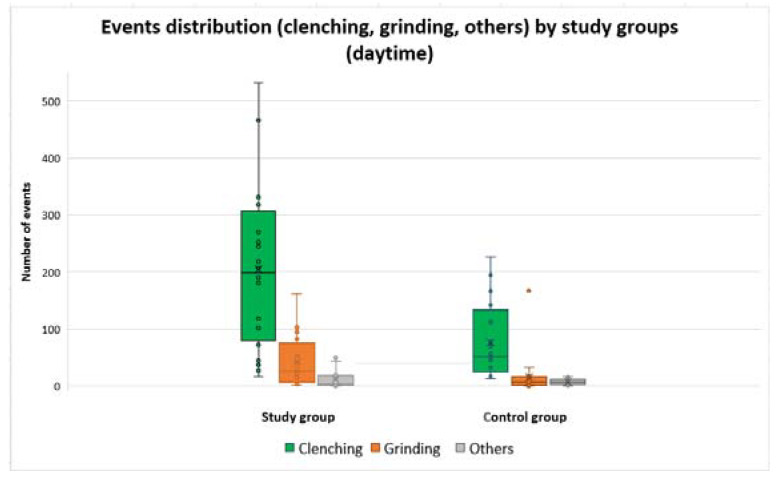
Distribution of clenching, grinding, and other types of events, for the two groups, during daytime.

The quartile distribution of these events is shown in Figure 3 and Figure 4. For the participants with SB in the study group, a higher frequency of clenching type events (tonic contractions) was noted, but was comparable in the range of values to grinding type events (phasic contractions).

The control group, compared to the study group, had fewer recorded events both during the day and at night.

### 3.4. Assessment of the Effort Made during Bruxism Activity (BWI)

BWI in SB for the study group had values between 0.134% and 1.283%, with a mean of 0.470%, compared to the control group which had values between 0.000% and 0.294% with a mean of 0.098%, the differences between the groups being statistically significant (Table 4).

BWI in AB for the study group had values between 0.094% and 4.480% with a mean of 1.177% compared to the control group which had values between 0.078% and 0.853% with a mean of 0.310%, the differences being statistically significant (Table 4).

### 3.5. Duration of Bruxism-Specific Activity (BTI)

BTI in SB for the study group had values between 0.294% and 3.236% with a mean of 1.055%, compared to the control group, which had values between 0.000% and 0.692% with a mean of 0.233%, the differences between the groups being statistically significant (Table 4).

BTI in AB for the study group had values between 0.279% and 10.103% with a mean of 2.788%, compared to the control group, which had values between 0.239% and 1.720% with a mean of 0.721%, the differences were statistically significant (Table 4).

### 3.6. Evaluation of the Personal Bruxism Index (BPI)

In the study group, the BPI value varied from 0.240% to 2.542% for SB with a mean of 0.860% and from 0.217% to 8.230% for AB with a mean of 2.251% (Table 5).

The BPI value in the control group ranged from 0.000% to 0.560% for SB with a mean of 0.188% and from 0.187% to 1.431% for AB with a mean of 0.585%.

In addition to these bruxism parameters, the device provided a series of data points related to the activity of the masseter muscle.

MWI in SB for the study group had values between 0.375% and 2.440% with a mean of 0.964%, compared to the control group which had values between 0.097% and 0.625% with a mean of 0.328%; the differences among the groups were statistically significant (Table 6).

MTI in SB for the study group had values between 1.265% and 9.612% with a mean of 3.351%, compared to the control group which had values between 0.580% and 2.471% with a mean of 1.318%; the differences between the groups were statistically significant (Table 6).

The MPI in SB for the study group had values between 0.968% and 7.221% with a mean of 2.555%, compared to the control group which had values between 0.419% and 1.831% with a mean of 0.988%; the differences between the groups were statistically significant (Table 6).

MWI in AB for the study group had values between 1.571% and 11.693% with a mean of 4.737%, compared to the control group, which had values between 1.565% and 4.339% with a mean of 2.772%, the differences between the two groups being statistically significant (Table 6).

MTI in AB for the study group had values between 6.747% and 31.270% with a mean of 17.015%, compared to the control group, which had values between 5.909% and 15.246% with a mean of 11.830%, the differences among the groups were statistically significant (Table 6).

MPI in AB for the study group had values between 5.021% and 24.745% with a mean of 12.923%, compared to the control group, which had values between 4.461% and 11.320% with a mean of 8.810%, and the differences that were recorded between those two groups were statistically significant (Table 6).

**Figure 5:**
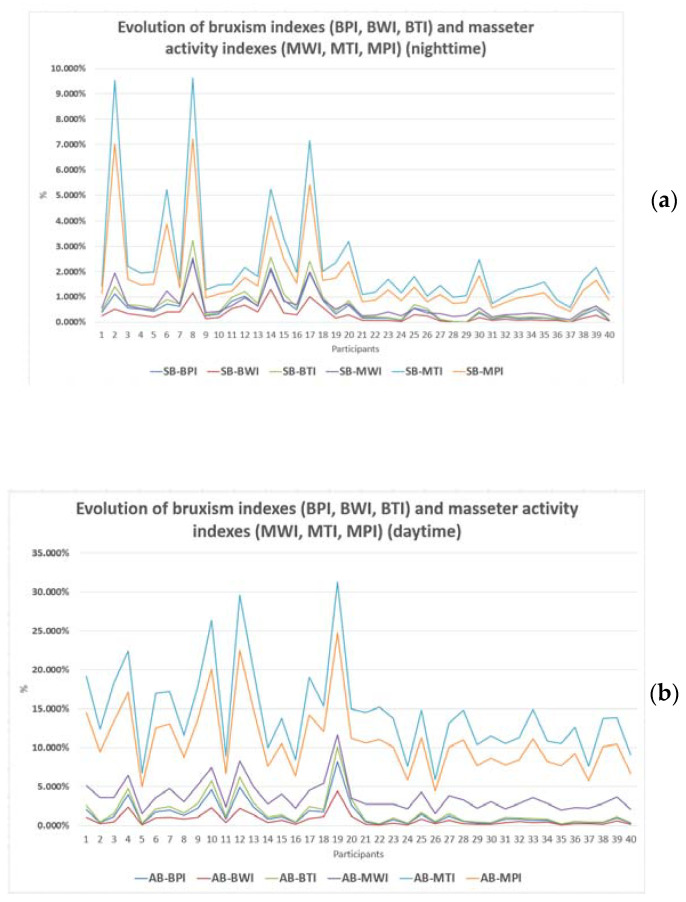
Evolution of the values of the indices for bruxism and for the activity of the masseter muscle: (**a**) sleep state and (**b**) awake state.

The overall values of bruxism and masseter muscle indexes are grouped in Figure 5a (for sleep state) and Figure 5b (for awake state). The first 20 sets of values correspond to the study group, while the other 20 sets of values correspond to the control group.

Apart from the above parameters, clinical signs representing manifestations of bruxism were also collected based on self-report.

Thus, 60% of the participants from the study group presented tooth wear compared to 15% of the control group, 10% of the participants in the study group presented abfraction lesions compared to 0% in the control group, 85% of the participants in the study group had masticatory muscle pain compared to 0% for the control group, and 35% of the study group participants had headaches and neck muscle pain compared to 0% for the control group.

Centralization of the data revealed statistically significant differences present between the study group and the corresponding control group (*p* < 0.05) in tooth wear, masticatory muscle pain, headache and neck muscle pain, and snoring (Table 7).

Among the data exported by the device, the BPI represents the most relevant index for the assessment of bruxism in both groups of participants (the differences between the groups are statistically significant).

Regarding the classification of bruxism based on to the circadian rhythm, based on the BPI assessment for SB and AB, the classification based on self-report was confirmed for the participants in the study group (14 participants in the study presented AB, 4 participants presented SB, and 2 participants presented a combined form with similar BPI values for SB and AB).

After sorting the 40 participants from the 2 groups, in descending order of BPI, it was found that 3 participants in the control group had BPI values comparable to those in the study group. The subsequent analysis of the questionnaires (from the previous study) and the clinical examination revealed that one of the participants reported a low level of stress, manifestations of temporomandibular disorder and difficulties in initiating sleep (rarely); the second participant, following the clinical examination, was found to have hypertrophy of the masticatory muscles and tooth wear; and the third, after the clinical manifestations of bruxism were explained to him, realized that he clenches his teeth during the day. According to the new assessment, these participants could be assigned to the “definite bruxism” study group.

For all 20 participants in the study group, sEMG recordings confirmed bruxism-specific sEMG activity, and those participants could be classified as “definite bruxism”. In addition, from the control group, three participants presented specific sEMG aspects of bruxism, being able to be assigned to the “definite bruxism” group. Consequently, the self-report was not always relevant.

## 4. Discussion

This research is in line with the current recommendations regarding obtaining a standardized tool used to assess bruxism, respectively, a multidimensional bruxism assessment system (STAB) [7,8,9,10,12], which is an extension of a study carried out on 328 students from the Faculty of Dental Medicine, University of Medicine and Pharmacy of Craiova, Romania [15] in 2021. In this study, based on the answers received from a questionnaire, the diagnosis of “possible bruxism” was established (SB, AB, or combined) for a total of 129 students. From the 129 students with “possible bruxism”, the study group consisting of 20 participants was selected, and from the 199 students without self-reported bruxism, the control group was also made up of 20 participants.

The best standard for the assessment of muscle activity in SB is represented by polysomnographic recordings, however, this investigation cannot currently be used in healthy individuals [16,17,18,19], having applicability only for research projects [20].

To confirm the diagnosis of “definite bruxism” and to be able to differentiate the activity carried out by the masseter muscle corresponding to participants with bruxism from the activity of those without bruxism, sEMGs of the masseter muscle were performed in both groups of participants for 24 h in the natural environment, respecting their daily routine. Electromyography is a technique for measuring, recording, and analyzing the myoelectric signal values which reflect the muscle activities [21]. Surface electromyography (sEMG) is frequently employed to measure and to monitor the activity of the masticatory and facial muscles [22] and may emphasize pathological and physiological states of the masticatory system. Surface electromyography is a useful tool in the diagnostic process, providing an accurate and predictable assessment of muscles’ activity [23].

The increasing usage of sEMG, both for research and clinical purposes, has demonstrated that recordings can be easily performed for both patient and physician [24]. sEMG signals are acquired by surface electrodes that measure electrical values at tissue level and indicate the temporal and spatial addition of the multitude of neighboring motor units [25]. sEMG is applied in the diagnosis process of patients that suffer from general muscle disorders [26,27,28,29,30], neuromuscular conditions, or diseases which influence the neuromuscular performance [23]. sEMG presents several applications in dentistry, more specifically, orthodontics, implantology, occlusology, temporomandibular disorder, and sleep disorders [31,32].

Making recordings in the natural environment of the patient was recommended in order to obtain the most relevant data regarding muscle activity from bruxism in the natural environment of the examined subject. Shiffman et al. [33] stated that, to record a behavior representative of a subject, it must be recorded in the natural environment where the event currently occurs. Several recent studies highlighted the applications of EMA in the study of AB or SB [7,34,35,36,37].

However, sEMG also has several disadvantages. First, the recordings’ analyses are limited to measuring the overall muscle activity, the interactions between different muscles, and the variability of the acquires signal values over time, through surface electrode detection of action potentials of overlapping motor units. One more significant disadvantage of sEMG is its susceptibility to impedance imbalance that can reduce the reliability level and reproducibility of EMG appraisal [23,24].

There are several ways to overcome this limitation: maintain a fair and constant gap between the electrodes, define a standard procedure indicating the placement of the surface electrodes, and perform an appropriate analysis of the sEMG recordings, from a quantitative point of view, relying on normalization procedures [31].

The dia-BRUXO device used for recordings was a wearable type and provided data regarding the number of specific bruxism events and their type, the time during which specific bruxism activities were carried out (BTI), the effort made by the masticatory muscles during the bruxism activity (BWI), personal bruxism index (BPI), data on masseter muscle activity, and recordings of EMG activity, but also a selection of representative activities. Data exported by the recording device included details of muscle activity in a resting position, tonic contractions, phasic contractions, mastication, swallowing, speech, teeth clenching, soft grinding, heavy grinding, and jaw thrusting [38,39]. The data analysis is carried out by studying the connection between the intensity and the frequency of the recorded electrical activity and the duration [40].

All these data represent physiological activities of the masticatory system and specific bruxism activities [7].

Wearable and portable devices that can be used for sEMG today are presented in a review by Yamaguchi in 2020 [41]. According to this study, 51 such devices with different performances are known. As the acquisition, recording, and processing of data vary between sEMG devices, the results could not be compared to other studies. We consider that our research is in line with the STAB directive that emphasized bruxism evaluation methods. The use of wearable devices for sEMG recordings of the masticatory muscles became more and more frequent, due to the patients’ preference to perform these recordings in their natural environment, in order to maintain their stress level and sleep schedule [41]. Prasad et al. [42] compares the results of EMG recordings performed in the lab with those performed in the patients’ natural environment, and they concluded that the differences regarding the amplitudes of the masseter muscles’ contractions were small and certainly not relevant from a clinical point of view (0.94–1.00 up to 0.82–1.00, respectively). Therefore, sEMG recordings represent a reliable evaluation method of the muscular activity in bruxism [42,43].

The recommendations of the experts who developed the consensus regarding STAB are for the use of such devices for the study of bruxism [12]. Additionally, ambulatory electromyographic (EMG) devices are more and more employed in SB studies. Methods regarding EMG signal acquisition, further processing, and evaluation techniques vary between studies [44]. This may affect the evaluation of bruxism, as well as the possibility to perform comparisons between various studies. It is the reason why experts in this field recommend a standardized way to report recording procedures [9,10,44].

Therefore, the results obtained following our study cannot be compared with those of other studies, because these recordings must be reported at a threshold of activity considered normal. This threshold may be considered as a proportion of the maximum level of voluntary contraction, as a multiple of the baseline level of muscle relaxation, or as the level of muscle activity reached during swallowing. Extensive variability has been found in the literature for the thresholds used to characterize SB events [44,45,46,47,48,49,50,51]. According to the manufacturer of the dia-BRUXO device, in this study, the events recorded for this device are reported relative to the maximum contraction.

Researchers are continuously seeking out valid and accurate diagnostic tools, especially since there is the question of when bruxism can be considered pathological and when it can be considered a normal behavior (normo- and patho-bruxism) and whether there is the need of treatment [17,52,53]. Thus, there are a few studies in literature about the applications of sEMG in bruxism, but the results differ greatly. Monteiro, in 2021, performed an sEMG study of the temporal muscle using a portable device called Myobox (NeuroUp, Brazil) in people with AB to establish the pattern of muscle contractions [43]. Thus, according to his study, 32.3% presented the tonic subtype, 16.8% presented the phasic subtype, and 50.8% presented an intermediate subtype. In the presented study in the participants with AB, the tonic contractions had the greatest weight, followed by the phasic ones and then by the intermediate ones. The different results can be attributed to the different ways of recording and reporting.

Another study by Lan KW et al. in 2022 evaluated, through EMG, the masticatory muscles’ activity in different types of bruxism, referring to centric and eccentric bruxism [45].

When comparing data between studies, consideration should be given to the diagnostic systems used to assess bruxism, the type of diagnosis used as a criterion (“possible bruxism”, “probable bruxism”, “definite bruxism”), the sample size, and whether the evaluation by different methods was performed simultaneously or at different times.

Without comparing the results obtained in this research with other studies, higher values of BTI, BWI, and BPI were found for all participants in the study group with “possible bruxism”; they can be considered as having the diagnosis of “definite bruxism”. Increased values of these bruxism indexes were also revealed in three participants from the control group. Clinically reassessing and discussing with these participants concluded that they presented signs of bruxism. In 2018, Lobbezoo et al. [7] emphasized the limitation of self-reporting regarding bruxism’s presence and also recommended the improvement on self-reporting methods that wound increase the reliability and precision of these methods.

The evaluation of the participants from this study also revealed a series of clinical signs specific to both bruxism and tooth wear, as well as signs common to bruxism and temporomandibular disorder: pain of masticatory muscles, headache and neck pain, and disorders of TMJ. Tooth wear was present in 60% of the study group participants compared to 15% from control group participants, with statistically significant differences. Manfredini et al. [54] conducted an EMG-based study in which he tried to correlate masseter muscle activity in SB with tooth wear, but the results demonstrated that it is not possible to use tooth wear as an indicator of SB or masseter muscle activity. Another study carried out on children aged 6–11 years using the factorial analysis method emphasized the fact that there is no correlation between bruxism and tooth wear in the evaluated children [55]. Among the clinical signs associated with bruxism, no abfraction lesions have been reported, but they represent, after all, also a form of tooth wear particularly specific to old age [56], as well as temporomandibular disorder; although it was not reported, it refers to two other clinical signs that have been reported (masticatory muscle pain and headache and neck muscle pain) [57,58].

Temporomandibular disorder is frequently associated with bruxism, but it is still not clear if bruxism is a manifestation of the temporomandibular disorder or of bruxism; through its excessive activity of the masticatory muscles, it determines the temporomandibular disorder. Manfredini performed a study in two clinical facilities, and he analyzed the dysfunction signs and self-reported bruxism [54]. However, he introduced new and modern investigation methods such as magnetic resonance imaging and polysomnography, and these substantially modified the results of the study, such that a correlation could no longer be established nor analyzed.

In a review published by Manfredini in 2010 [59], the association between bruxism and the temporomandibular disorder was also analyzed, and the final conclusion was that a correlation is unlikely to exist. In 2006, Feteith [60] performed a small study on young adolescents, indicating the simultaneous presence of TJM and bruxism, while Anastassaki [61], in 2004, performed a study on 3196 patients. His final conclusion was that all symptoms of the TJM in adults, excepting crepitations, are associated with a conscientization of teeth clenching and grinding.

The use of sEMG to analyze the overall activity corresponding to the masseter muscle in participants with bruxism is part of the STAB systems, axis A [10,11,12,14]; it is easy to perform, it is highly accepted by participants with possible bruxism, and provides more than enough data to confirm the diagnosis of bruxism.

The limits of the present study are given by the number of participants and the duration of recordings. It should also be emphasized that the present research provided the frequency, duration interval, and the intensity of masticatory muscle activity in two groups of participants: a group with “possible bruxism” and a group that reported no signs of bruxism. The data recorded on the EMG dia-BRUXO device represent separated entities from those recorded by other devices and PSG recordings.

## 5. Conclusions

Based on data provided by the dia-BRUXO device, the sleep duration of participants from the study group was reduced compared to the sleep duration of participants from the control group, confirming the findings from our previous study, according to which participants with bruxism have difficulties initiating and maintaining sleep.

Participants in the study group with AB showed predominantly clenching type events (tonic contractions), and those with SB showed clenching type events (tonic contractions) and grinding-type events (phasic contractions) in comparable proportions. All device-reported bruxism indices (BWI, BTI, BPI, MPI, MWI, MTI) had higher values in the study group compared to the control group, confirming greater activity in terms of masticatory muscle effort and activity duration. The BPI index is the most important parameter exported by the dia-BRUXO device; it is calculated based on BWI and BTI. Ordering the BPI values in a descending order indicated that all participants in the study group with “possible bruxism” showed EMG activity of the masseter muscle specific to bruxism and can be classified as “definite bruxism”, and three participants in the control group presented BPI values comparable to those of the participants in the study group and can be classified as “definite bruxism”.

Among the clinical signs accompanying bruxism, tooth wear, masticatory muscle pain, headache and neck muscle pain, and snoring were confirmed in the study group.

Bruxism represents an interesting scientific topic for experts in dentistry, sleep medicine, and the study of myofascial pain. The identification of an easy and reliable method to examine the activity of the masseter muscles in patients with bruxism is justified by the increase in the prevalence of this disorder as well as its clinical manifestations.

## Figures and Tables

**Table 1:** Demographic characteristics of the two groups.

	Value	Study Group (N = 20)	Control Group (N = 20)	*p* *
Age	Minimum	20	21	-
	Maximum	35	53	-
	Mean ± SD	25.4 ± 3.74	28.6 ± 8.76	0.398
Gender	Females	15 (75%)	15 (75%)	-
	Males	5 (25%)	5 (25%)	-

* Mann–Whitney U test.

**Table 2:** Asleep and awake state duration and device detachment time.

dia-BRUXO Parameter	Study Group			Control Group			*p*
	Min	Max	Mean ± SD	Min	Max	Mean ± SD	
Sleep duration (%)	24.000	48.900	34.790 ± 7.670	19.200	50.700	30.485 ± 8.600	0.103 *
Awake duration (%)	51.100	76.000	64.615x ± 7.993	49.300	80.800	69.515 ± 8.600	0.070 *
Detachment duration (%)	0.000	17.400	1.430 ± 4.152	0.000	7.100	0.530 ± 1.633	0.058 **

* Mann–Whitney U test; ** Independent samples *t*-test.

**Table 3:** SB and AB events.

dia-BRUXO Parameter	Study Group			Control Group			*p*
	Min	Max	Mean ± SD	Min	Max	Mean ± SD	
SB—Clenching	9	96	37.850 ± 23.374	0	37	11.050 ± 11.133	<0.005 *
SB—Grinding	2	201	39.950 ± 50.784	0	21	5.150 ± 5.696	<0.005 *
SB—Others	0	127	9.850 ± 28.380	0	4	0.500 ± 1.277	0.008 *
AB—Clenching	17	533	204.250 ± 143.934	13	227	76.250 ± 65.240	0.002 *
AB—Grinding	1	162	42.750 ± 45.163	0	33	8.350 ± 9.155	0.002 *
AB—Others	0	50	11.600 ± 14.486	0	17	7.100 ± 5.261	0.860 *

* Mann–Whitney U test.

**Table 4:** BWI and BTI indexes in SB and AB.

dia-BRUXO Parameter	Study Group			Control Group			*p*
	Min	Max	Mean ± SD	Min	Max	Mean ± SD	
SB—BWI (%)	0.134	1.283	0.470 ± 0.330	0.000	0.294	0.098 ± 0.085	<0.005 *
SB—BTI (%)	0.294	3.236	1.054 ± 0.790	0.000	0.692	0.233 ± 0.200	<0.005 *
AB—BWI (%)	0.094	4.480	1.177 ± 1.028	0.078	0.853	0.310 ± 0.214	<0.005 *
AB—BTI (%)	0.279	10.103	2.788 ± 2.380	0.239	1.720	0.721 ± 0.421	<0.005 *

* Mann–Whitney U test.

**Table 5:** BPI index in SB and AB.

dia-BRUXO Parameter	Study Group			Control Group			*p*
	Min	Max	Mean ± SD	Min	Max	Mean ± SD	
SB—BPI (%)	0.240	2.542	0.860 ± 0.632	0.000	0.560	0.188 ± 0.162	<0.005 *
AB—BPI (%)	0.217	8.230	2.251 ± 1.925	0.187	1.431	0.585 ± 0.350	<0.005 *

* Mann–Whitney U test.

**Table 6:** MWI, MTI, and MPI indexes in SB and AB.

dia-BRUXO Parameter	Study Group			Control Group			*p*
	Min	Max	Mean ± SD	Min	Max	Mean ± SD	
SB—MWI (%)	0.375	2.440	0.964 ± 0.620	0.097	0.625	0.328 ± 0.131	<0.005 *
SB—MTI (%)	1.265	9.612	3.351 ± 2.620	0.580	2.471	1.318 ± 0.469	<0.005 *
SB—MPI (%)	0.968	7.221	2.555 ± 1.940	0.419	1.831	0.988 ± 0.353	<0.005 *
AB—MWI (%)	1.571	11.693	4.737 ± 2.372	1.565	4.339	2.772 ± 0.716	0.001 *
AB—MTI (%)	6.747	31.270	17.015 ± 6.725	5.909	15.246	11.830 ± 2.761	0.005 *
AB—MPI (%)	5.021	24.745	12.923 ± 5.231	4.461	11.320	8.810 ± 2.026	0.004 *

* Mann–Whitney U test.

**Table 7:** Clinical signs representing manifestations of bruxism.

Parameter	Value	Study Group (N = 20)	Control Group (N = 20)	*p* *
Tooth wear	No	8 (40%)	17 (85%)	**0.003**
	Yes	12 (60%)	3 (15%)	
Abfractions	No	18 (90%)	20 (100%)	0.244
	Yes	2 (10%)	0 (0%)	
Masticatory muscle pain	No	3 (15%)	20 (100%)	**<0.005**
	Yes	17 (85%)	0 (0%)	
TMJ disorders	No	15 (75%)	19 (95%)	0.077
	Yes	5 (25%)	1 (5%)	
Headache/neck pain	No	13 (65%)	20 (100%)	**0.004**
	Yes	7 (35%)	0 (0%)	
Interocclusal appliance	No	17 (85%)	20 (100%)	0.115
	Yes	3 (15%)	0 (0%)	
Snore	No	14 (70%)	19 (95%)	**0.046**
	Yes	6 (30%)	1 (5%)	

* Chi-Square/Fisher Exact Test.

## Data Availability

The authors declare that the data of this research are available from the correspondence authors upon reasonable request.
